# Supplementary material for: Hypocalcaemia and calcium intake in pregnancy: A research protocol for critical analysis of risk factors, maternofoetal outcomes and evaluation of diagnostic methods in a third-category health facility, Cameroon
Source: PLoS One. 2020 Nov 5;15(11):e0241812. doi: 10.1371/journal.pone.0241812 (PMC7644052; doi:10.1371/journal.pone.0241812)
Supplement: S1 Data — (DOCX) [file pone.0241812.s001.docx]

# S1 Data. Research questionnaire /data collection tool

Participant identification code:…………………………………………….. Date:……………………………………….

Name of data collector:……………………………………………………………

Section 1: Characteristics of participants

1. Age of participant _____________________________________________________
2. Marital status: a)Married b) Single c)Divorced d) Widow
3. Religion: a)Catholic b)Protestant c)Presbyterian d) Muslim e) Atheist f)__________________
4. Level of education of participant: a)Never schooled b)Primary c)Secondary d)Higher e)Koranic
5. Level of education of Partner: a)Never schooled b)Primary c)Secondary d)Higher e)Koranic
6. Average number of people in your household_________________________________
7. Estimated monthly income: a)Less than 50T b)50-100T c)100-200T d) More than 200T
8. Number of antenatal consultations: a) None b) 1-3 c) 4-6 d) Above 6
9. Gestational age at booking ANC____________________________
10. Total number of pregnancies______________, Number of deliveries__________, Number of children alive_____________, Average spacing between pregnancies_________, Duration of last breastfeeding___________________________
11. Age of last child________________________________________________________
12. Past history of hypertension in previous pregnancies? a) Yes b)No
13. Have you ever had a miscarriage? a) Yes b)No. If yes how many?____
14. Have you had a stillbirth before? a) Yes b)No, If yes , how many?_______

Section 2: Nutritional habits

1. How many square meals were you taking daily before onset of pregnancy? __________
2. How many square meals do you take daily during pregnancy?_____________________
3. What is the major content of your most consumed meal? a) Rice b) Tubers (sweet potatoes, cocoyams, yellow yam, cassava etc) c)Banana and plantains d) Corn e)others
4. At what time do you take fruits? a) Before meals b) During meals c)After meals d)between meals
5. What fruits do you usually consume most? a) Ripe banana b) Watermelon c)Oranges d) Pineapple e) Others____________________________________
6. How often do you associate legumes/vegetables to your meals? a) Never b)Occasionally c) 1-2 times a week d)3-5 times a week e) Everyday
7. Do you take any other deserts between meals? a) Yes b) No.
8. How often do you take tea/with milk? a) Never b)Occasionally c) 1-2 times a week d)3-5 times a week e) Everyday
9. How often do you take coffee/with milk ?: a) Never b)Occasionally c) 1-2 times a week d)3-5 times a week e) Everyday
10. How often do you take cheese? a) Never b)Occasionally c) 1-2 times a week d)3-5 times a week e) Everyday
11. How often do you take cake in any of its forms? a) Never b)Occasionally c) 1-2 times a week d)3-5 times a week e) Everyday
12. How often have you been taking “calabar chalk” during your pregnancy? a) Never b)Occasionally c) 1-2 times a week d)3-5 times a week e) Everyday
13. What is your main drinking water source? a) Treated wells b) Camwater c) Mineral water d)Streams e)Others_______________________________

Section 3: Calcium supplementation

1. At what age of pregnancy did you start antenatal consultations? a) 1-2 months b) 2-3 months c) 4-6months d) above 7 months.
2. Did you take calcium supplements during your pregnancy? a) Yes b) No
3. If yes, for about how many moths in pregnancy? a) 1-3 months b)4-5 months c) 6-7 Months d)all through pregnancy
4. What dose of elemental calcium were you taking a day? a) 300mg-500mg b)500mg-600mg c)1000mg d)1000mg-1500mg e)2000mg.
5. Calcium prescription: a) By personnel b) auto-prescription c) On request by the pregnant woman
6. Have you been advised by health personnel on the necessity to take calcium supplements? a)Yes b)No
7. Why is it important to take calcium supplements in pregnancy? a) I don’t know b) For foetal bone development and growth c) to prevent cramps in the mother d) prevent high blood pressure in pregnancy, e)others
8. Did you take iron and folic acid supplements in pregnancy? a) Yes b) No , c)I took only folic acid, e)I took only iron supplements.
9. If yes, How often? a) Everyday b) At least once every two days c) Rarely
10. Do you take calcium supplements at the same time as the iron and or folic acid supplements? a) Yes b)No c) I don’t take calcium supplements
11. Does your partner support you and remind you on the need to take your supplements? a) Always b)Sometimes c)Occasionally d) Never
12. Who pays for your supplements? a) I pay for them b)My partner c)My parents d)Others______________________

Section 4: Maternal/Neonatal parameters and assays

Neonatal parameters

1. Duration of active phase of labor in hours____________
2. Apgar Score first minute______________________ Apgar score 5^th^ minute___________
3. Foetal Birthweight (FBW) in grammes___________Head circumference(HC) in cm____
4. Brachial Circumference (BC) in cm___________ Length of the baby in cm ___________________
5. Neonatal resuscitation: a) Yes b) No

Maternal parameters

1. Weight of woman in postpartum in kg_______________ Height of woman___________
2. BP at booking visit: Diastolic BP________________, Systolic BP________________
3. Gestational age at delivery___________________________________________
4. Systolic blood pressure_____________________ Diastolic blood pressure ___________

Blood assays

1. Total Calcium levels by atomic absorption spectrophotometry______________________
2. Total albuminemia by atomic absorption spectrophotometry_____________________
3. Albumin-corrected calcium levels ______________________________
4. Ionised calcium level by Ion-selective electrode potentiometry (ISEP)__________________
