# Supplementary material for: Hypocalcaemia and calcium intake in pregnancy: A research protocol for critical analysis of risk factors, maternofoetal outcomes and evaluation of diagnostic methods in a third-category health facility, Cameroon
Source: PLoS One. 2020 Nov 5;15(11):e0241812. doi: 10.1371/journal.pone.0241812 (PMC7644052; doi:10.1371/journal.pone.0241812)
Supplement: Attachment — Submitted filename: RESPONSE TO REVIEWERS COMMENTS.docx [file pone.0241812.s002.docx]

**RESPONSE TO REVIEWERS COMMENTS (PONE-D-20-07598R1)**

**Dear reviewers/editors,**

We wish to thank you all for the time put in to make changes on this research protocol. All recommended revisions have been made and the protocol resubmitted for re-evaluation.

| Comments | Response |
| --- | --- |
| **Reviewer 1** | |
| Congratulation and wish you the best for coming project. A minor mistake is that you are measuring blood lead level not "serum lead level". This is repeated 19 times in the MS. Please change them accordingly. | Thanks for your review and comment. We have considered changing the word serum to blood |
| **Reviewer 2** | |
| - While interesting, the 1st paragraph is unnecessary. | Thanks for the observation. The first paragraph been taken off |
| - The inclusion/exclusion criteria for the study is very vague in it's current form. What were the age ranges? Were women of all gravidas included? Was BMI considered, or a covariant as this could affect calcium absorption/metabolism? | Thanks for the observation. As stated in our protocol, any pregnant woman will be included whatever be her age, gravida, BMI. All these are important independent variables which would be measured to see their effect on hypocalcaemia in pregnancy. Most if not all potential causes of hypocalcaemia have been excluded by evaluation of past medical history and medical records. |
| - It is not necessary to show the formular for the sample size calculation. | Thanks for your observation. The formula has been taken off. |
| - The objectives might be better understood/visualized if they were presented in a table | Thanks for the observation. We have modified practically all of the introduction to help justify the main objectives of this study. We however do not understand when the reviewer recommends a presentation on a table. |
| - What is the potential scientific/clinical impact of this study? | The potential scientific impact of this study has been presented on page 15/16 |
| **Reviewer 3** | |
| **Major comments** | |
| 1. Pg. 2, ln. 25: Would not total calcaemia and albumin-corrected calcaemia underestimate the burder of hypocalcaemia, instead of overestimate? Because if most of pregnant women were diagnosed with hypocalcaemia, I assume that calcium supplementation would be routinely prescribed by health care providers and this would most likely not be an issue. | Thanks for your question. We actually think using ionized calcaemia to define hypocalcaemia will give us a true picture of the problem. We expect that the true prevalence of this problem be lower than what is reported in literature. But we at the same time think that even if rates are lower than what is currently presented, the impact remains great. Calcium supplementation has been recommended by the WHO in our context since 2013 but we persist with these high rates. It is for this reason that in trying to clearly define the burden, fully evaluate calcium supplementation and its barriers and find the role of effective supplementation on calcaemia. All this is coupled to evaluation of nutritional variables which are not only calcium sources but which could be the factor reducing calcium absorption even when calcium supplementation is effective. |
| 2. Pg. 2, ln. 33-34; pg. 6, ln. 128-129; pg. 7, ln. 142-146; pg. 8, ln. 172-174; pg. 14, ln. 326-330: I am intrigued how this study will address this objective with the current design. Apparently healthy pregnant women will be enrolled in this study as they arrive the maternity for their last prenatal visit (equal or greater than 37 weeks of gestation). Thus, the chances for the enrolled women to develop pregnancy-induced hypertension in just 1-3 weeks are relatively low. Related to this, if pregnant women present with high blood pressure during enrollment and fulfilling of questionnaire survey, they will not be healthy but hypertensive. Please clarify these issues. | Thanks for your questions. We understand that to meet this objective, this is not the ideal design. But our design is a classic design to establish strong hypotheses for verification with stronger experimental designs. In essence, there is no follow-up period in our study. We are obliged to see these women twice. The first time for the collection of data in the questionnaire, maternal parameters and collection of blood samples. We only then meet the woman again at delivery for foetal parameters and delivery outcomes. We avoided collecting all this data in labor or during delivery because labor and the high activity of the woman could have an impact on her blood calcium levels. As concerns hypertension, we are interested in finding the proportion of pregnant women with hypocalcaemia who developed hypertension of any form in their pregnancy. Women who had chronic hypertension before pregnancy will be excluded in analyzing for this objective. This is a cross sectional design which will help us develop stronger hypotheses for testing. To avoid this confusion, our study designed has been stated as cross sectional (no more nested with a cohort design) in which we are obliged to see the woman before and after delivery. |
| 3. Pg. 5, ln. 84: I recommend rewriting the following sentence: “Somewhat higher maternal lead levels have been linked to reduced fetal growth.” This might occur because calcium and lead compete for intestinal absorption, resulting in reduced maternal circulating levels of calcium which are necessary for fetal growth. There are several articles published in this area. Please briefly discuss and cite them. | Thanks for the recommendation. This has been modified and discussed. See Page 4/5, line 74-86 |
| 4. Pg. 6, ln. 124-131: Please number these objectives to match the description in the sub-section “Sampling and Sample number” in the article. | Thanks for your suggestion. The objectives have now been numbered. See page 6. |
| 5. Pg. 6, ln. 124: Please add which determinants (demographic, socio-economic, nutritional, etc.) you will examine. | Thanks for your recommendation. This has been added to objective 1. See page 6. |
| 6. Pg. 12, ln. 254: Please clarify what “booking antenatal visit” means. | Thanks for the worry. The booking ANC is the first ANC of the woman. This will help us get baseline parameters (principally the baseline blood pressure) of the woman to be able to know if she has experienced a significant increase with increasing gestational age. These precisions have been added. See page 11. |
| 7. Pg. 17, ln. 376: Will blood copper levels be measured in this study as well? If yes, you should provide the rationale and methodology for measuring it in this article. | Thanks for the observation. Blood copper levels will not be measured in this study. The typing error has been corrected. |
| 8. General: The English language of the entire article needs to be slightly improved, especially regarding verb conjugation. | Thanks for the observation. We noticed a series of language errors that have been addressed in the revised version. |
| **Minor comments** | |
| 1. Pg. 2, ln. 18-19: Please delete the words “among pregnant women”.  2. Pg. 2, ln. 23: Please change the word “means” for “mean”.  3. Pg. 4, ln. 62-63: Please delete the words “in pregnancy”.  4. Pg. 4, ln. 66: Please add the word “concentration” between “skeleton” and “during”.  5. Pg. 5, ln. 100: Please change the words “author in his work” for “authors”. | Thanks for your corrections. These comments have been addressed and integrated into this revised version. |
| 6. Pg. 6, ln. 114: Please add the word “compared” between “pregnancy” and “with”.  7. Pg. 6, ln. 131: Please add the word “compared” between “pregnancy” and “with”.  8. Pg. 7, ln. 149: Please add the name of the division.  9. Pg. 8, ln. 165: Will this equipment be provided by who?  10. Pg. 9, ln. 192: Please change the font of the letter “p” for lower case to match the formula. | Thanks for the corrections. These concerns have been addressed in the new version. |
| 11. Pg. 9, ln. 196: The words “Confidence level 95%” seem to be lost in the text.  12. Pg. 10, ln. 215: Please change the word “value” for “values”.  13. Pg. 10, ln. 228-230: These two sentences were already mentioned previously in the Materials and Methods section. Please delete them here.  14. Pg. 10, ln. 232: Please add the word “process” between “consenting” and “and”.  15. Pg. 10-11, ln. 237-239: Please change this sentence as following: Section 2 will contain specific questions regarding the nutritional behavior of the participants, while section 3 will be related to calcium supplementation and its co-variables. | Thanks for your corrections. All the comments have been addressed in the new version. |
| 16. Pg. 12, ln. 251-253: The information in this sentence is repeated below. Please incorporate these measurement devices to their respective sentences below.  17. Pg. 12, ln. 259: Please delete the word “included”.  18. Pg. 12, ln. 263: Please delete the words “on a digital weighing scale".  19. Pg. 12, ln. 264: Please spell out the abbreviation “BMI”.  20. Pg. 13, ln. 284: Please delete the words “immediately after birth”. | Thanks for the corrections. All these corrections have been incorporated. |
| 21. Pg. 13, ln. 287: Please change the word “ten” for “a scale of 0-10”.  22. Pg. 13, ln. 294: Please change the word “equations” for “equation”.  23. Pg. 15, ln. 336-337: Please delete the words “calcium content of meals”, since it is mentioned in pg. 17, ln. 386-388 that the calcium content of meals will not be measured in this study.  24. Pg. 17, ln. 399; pg. 18, ln. 415 and 424: Please change the word “risk” for “risks”.  25. Pg. 19: Please delete the last word “and” in “Ethical evaluation and approval, Acquisition of research materials and equipment, Training of data collectors and”. | Thanks for the corrections. These corrections have been addressed in the new version. |
