# Supplementary material for: Hypocalcaemia and calcium intake in pregnancy: A research protocol for critical analysis of risk factors, maternofoetal outcomes and evaluation of diagnostic methods in a third-category health facility, Cameroon
Source: PLoS One. 2020 Nov 5;15(11):e0241812. doi: 10.1371/journal.pone.0241812 (PMC7644052; doi:10.1371/journal.pone.0241812)
Supplement: Attachment — Submitted filename: Review comments.docx [file pone.0241812.s003.docx]

**REVIEW COMMENTS**

Thank you for asking me to review this protocol titled: “Hypocalcaemia and calcium intake in pregnancy: a critical analysis of risk factors, maternofoetal outcomes and evaluation of diagnostic methods in a third-class health facility”

Hypocalcaemia in pregnancy remains a problem in the LMICs and every measure to tackle it and thus reduce the associated potential adverse maternal and foetal effects will be very helpful. Hence, I consider this study very useful.

I, however, have made some observations and will be worth addressing them by the authors to improve the quality of the study.

**Major comments**

1. The use of mean blood pressure reading from both arms is not always correct and can either underestimate or overestimate the actual blood pressure. It is only useful if the difference is less than or equal to 10 mmHg. Kindly refer to the American Heart Association guide for blood pressure check.
2. Are you estimating iCa in the serum or plasma? There seem to be some conflicts with this in your methodology. For this study looking at the burden of hypocalcaemia, serum estimation will be more appropriate, and the methodology should support this if that is the case. The use of heparinized bottles overestimates hypocalcaemia even when calcium titrated heparin bottles are used. Not just from the heparin binding the calcium but from other blood components like the erythrocytes. There are several studies in support of this.
3. Change in blood pressure between booking and presentation of 30 mmHg systolic or 15 mmHg diastolic has long been removed from criteria for making diagnosis of hypertensive disorders in pregnancy. This is because there have been no associated adverse effects with this. Check the National High Blood Pressure Education Working Group report.
4. What is unique with a p value of <0.25 as criteria for inclusion in the multiple regression model? While not all variables or the significant ones in the bivariate analysis i.e. p<0.05?
5. Data management and analysis should be presented according to the objectives.
6. Your questionnaire suggest that you are assessing for levels of lead and copper. Include details of your assessment of lead and copper in your methodology and it needs to be appropriately justified in your introduction section.

**Minor comments**

1. “Term” begins at 37 completed weeks. The use of “near term” while your population comprise of women of gestational age 37 weeks and above will not be correct.
2. The terms “structured” and “semi-structured” questionnaire was used interchangeably multiple times in this writing. Looking at your questionnaire, I will advise you stick with “semi-structured” questionnaire.
3. The term “retarded” is an obsolete word to describe foetal growth. We talk of “foetal growth restriction” and not “foetal growth retardation”, that appeared severally in your writing.
4. Kindly re-write the first sentence of paragraph 3 of the introduction section as it is not clear in its current form.
5. While your topic will suggest that you aim to determine the materno-foetal outcomes of hypocalcaemia, your objective 3 suggest that you aim to determine the materno-foetal outcomes of hypertensive disorders in pregnancy. Kindly harmonise and rewrite.
6. Provide reference for Page 8, In. 160-162
7. The protocol will benefit from a review for coherence and conjunctions.
8. On your questionnaire, I have the following observations and comments:
a. I am not sure if the aim of Q10 was to assess parity. If it is, the number of deliveries will be more appropriate rather than number of children alive.
b. The purpose of asking for abortions (better term “miscarriages”) in Q13 is not quite clear from your protocol. However, I will suggest separating miscarriages from stillbirths/ENND.
c. Create opportunity for “others” in Q16 and Q33.
d. Separate iron and folic acid in Q34 as a woman may not be taking both.
e. Question 46 on your questionnaire is same as Q9. I will suggest including the gestational age at recruitment and at delivery as these are potential confounders.

Thank you.
