# Supplementary material for: Hypocalcaemia and calcium intake in pregnancy: A research protocol for critical analysis of risk factors, maternofoetal outcomes and evaluation of diagnostic methods in a third-category health facility, Cameroon
Source: PLoS One. 2020 Nov 5;15(11):e0241812. doi: 10.1371/journal.pone.0241812 (PMC7644052; doi:10.1371/journal.pone.0241812)
Supplement: Attachment — Submitted filename: RESPONSE TO REVIEWERS.docx [file pone.0241812.s005.docx]

RESPONSE TO REVIEWERS

We want to appreciate all the reviewers for their time sacrificed to raise very vital comments to better this protocol. The additional comment raised by reviewer three is addressed here.

**Comment:** Thank you for adressing most of my suggestions, however the main issue in methdology is still not answered. The title of your research include fetomaternal outcomes however, you excluded most of these outcomes from being looked for, I think you can either change your study to just assess risk factors of hypocalcemia and assessing its prevelance without searching for the outcomes Or you can include all pregnant women in the 3rd trimster admitted for labor and assess for hypocalcemia risk factor , calcium level, obtain your questionnaire that assess different habits and nutritional factors. If you choose the 2nd option I think you have to adjust the sample size to get statistically adequate results.

**Response:** We have a problem considering to recruit and measure calcaemia and other outcomes like BP earlier because;

1. Most often the first trimester and most of the second trimester is dedicated to improvement on the calcium stores of the mother in order to prepare for the high demand in the third trimester. We therefore wanted to measure these calcium levels only when we are sure that a great deal of foetal growth has occurred in the third trimester (1). This will help us evaluate to know whether the calcium stores the women get can really support them for their pregnancy till term (reason for which we considered term pregnancies). If we recruit earlier we will miss a great deal of women who will be susceptible to hypocalcaemia.

2. We could not consider collecting this data in labor and including every woman who presents in labor because, the hyperventilation and strain of the arms and uterine contractions are likely to affect serum calcium levels. In addition, measuring and getting adequate blood pressures in labor will be difficult and contrary to recommendations of standard BP measurement (2)(3).

Our current design can determine a major maternal outcome which is hypertensive disorder in pregnancy (from which a sample size calculation was done). We recognize that our inclusion gestational age will not allow us measure important foetal outcomes. We have included this as a limit to this protocol (page 16, first paragraph) and have considered to still evaluate these foetal outcomes but precise that they will be evaluated as secondary outcomes. We belief it will be a great waste if we have these babies at term and cannot find out if some of their outcome variables could be linked to hypocalcaemic states of the mother. We have difficulties to shift this time of recruitment of participants because of the two major reasons stated above.

References

1.	Kovacs CS. Maternal mineral and bone metabolism during pregnancy, lactation, and post-weaning recovery. Physiol Rev. 2016;96(2):449–547.

2.	Muntner P, Shimbo D, Carey RM, Charleston JB, Gaillard T, Misra S, et al. Measurement of blood pressure in humans: A scientific statement from the american heart association. Hypertension. 2019;73(5):E35–66.

3.	Jafri L, Khan AH, Azeem S. Ionized calcium measurement in serum and plasma by ion selective electrodes: Comparison of measured and calculated parameters. Indian J Clin Biochem. 2014;29(3):327–32.
